# Leverage biomaterials to modulate immunity for type 1 diabetes

**DOI:** 10.3389/fimmu.2022.997287

**Published:** 2022-11-02

**Authors:** Zhangyan Jing, Yuan Li, Yumeng Ma, Xiaozhou Zhang, Xin Liang, Xudong Zhang

**Affiliations:** ^1^ Department of Pharmacology, School of Medicine, Shenzhen Campus of Sun Yat-sen University, Sun Yat-sen University, Shenzhen, Guangdong, China; ^2^ Guangdong Provincial Key Laboratory of Medical Molecular Diagnostics, Key Laboratory of Stem Cell and Regenerative Tissue Engineering, School of Basic Medical Sciences, Guangdong Medical University, Dongguan, China

**Keywords:** biomaterial, type 1 diabetes, immune modulation, immune checkpoint, vaccine

## Abstract

The pathogeny of type 1 diabetes (T1D) is mainly provoked by the β-cell loss due to the autoimmune attack. Critically, autoreactive T cells firsthand attack β-cell in islet, that results in the deficiency of insulin in bloodstream and ultimately leads to hyperglycemia. Hence, modulating immunity to conserve residual β-cell is a desirable way to treat new-onset T1D. However, systemic immunosuppression makes patients at risk of organ damage, infection, even cancers. Biomaterials can be leveraged to achieve targeted immunomodulation, which can reduce the toxic side effects of immunosuppressants. In this review, we discuss the recent advances in harness of biomaterials to immunomodulate immunity for T1D. We investigate nanotechnology in targeting delivery of immunosuppressant, biological macromolecule for β-cell specific autoreactive T cell regulation. We also explore the biomaterials for developing vaccines and facilitate immunosuppressive cells to restore immune tolerance in pancreas.

## 1 Introduction

Type 1 diabetes (T1D) is a metabolic syndrome caused by the loss of autoimmune tolerance to insulin-producing islet β-cells (1). Pancreatic infiltrated islet-autoreactive T cells destroy the β-cells, resulting in insufficient insulin secretion, that elicits hyperglycemia (2). T1D is commonly occurred in children and adolescents with age from 1 to 15 years old, and around 1.2 million cases worldwide are diagnosed with T1D by 2021 (3). Moreover, the incidence of T1D is rising ever year. There are annually almost 30,000 new cases in United States and 13,000 new cases in China (3).

There have been many applications of immunotherapy for T1D. Currently, insulin replacement therapy is the main form of treatment for T1D since the discovery of insulin in 1921 by Frederick Banting and his colleague. Hereafter, pharmaceutical companies have developed a variety of insulin analogues such as fast-acting insulin and long-acting insulin analogues for the treatment of T1D (4). However, exogenous insulin injection cannot accurately mimic the physiological insulin secretion by the β-cells through sensing the high level of blood glucose (5). Thus, the careless of management of blood glucose could cause hyperglycemia, and long-term hyperglycemia may lead to many diabetic complications such as diabetic heart disease, diabetic neuropathy, diabetic nephropathy, etc. (4). On the other hand, excess insulin administration may also cause hypoglycemia followed by coma and even the risk of death (6). Therefore, glucose-sensing and responsive insulin delivery system represents an ideal treatment of T1D. Numerous strategies were proposed, such as glucose oxidase-based systems, glucose-binding molecule (particle)-based systems and phenylboronic acid-based systems, which were reviewed in detail before (7).

Islet transplantation is another alternatively treatment for T1D. Shapiro and colleagues successfully performed islet transplant on a patient firstly in 2000, and this standard procedure was known as “Edmon protocol” (8). Although islet transplantation can be technically achieved, it still faces many problems such as the lack of donors, cumbersome isolation of islets, immune rejection of transplantation, long-term need for immunosuppressants, and islet death caused by immune rejection and hypoxia (3). To conquer these issues, utilizing biomaterial to encapsulate islets, β-cells or induced pluripotent stem cells enhances our insight into better transplantation in remission of autoimmune responses, amelioration of hypoxia and protection against fibrosis. Various strategies, including modification of grafts, encapsulation of functional materials (e.g., can produce oxygen), co-transplantation with other cells or molecules, offer an attractive modality.

T1D is considered as a T cell-mediated autoimmune disease, and involves in both of innate immune and acquired immune cells (9). Critically, the abnormality of peripheral immune tolerance system such as the loss of regulatory T cells (Tregs) and decrease of programmed cell death-ligand 1 (PD-L1) also leads to T1D (10). Thus, inhibiting the immune activity and restoring self-tolerance to pancreatic β-cells are novel treatment strategies for T1D. Immunosuppressive agent cyclosporine A were firstly used in the study of treatment for T1D (11). Although cyclosporine A could protect β-cells, its nephrotoxicity led to the termination of the studies (11). Next, anti-CD3 monoclonal antibody was utilized to deplete T cells to avoid autoimmune attack the residual β-cells, that could permanently reverse the diabetes in the NOD mice (12). Regardless of anti-CD3 monoclonal antibody may also cause the risk of infection, it is still under the study of phase III clinical trial, and may become an potent antibody drug for T1D treatment (13). T cell immune checkpoints are a class of receptors present on the surface of T cells, or intracellular metabolic enzymes that regulate T cell activity, such as programmed death-1 (PD-1), T cell immunoglobulin and mucin domain-containing protein 3 (TIM-3), and lymphocyte activation gene-3 (LAG-3) (14). Many types of cancer cells abnormally express immune checkpoint inhibitory ligands such as PD-L1, enabling cancer cells to escape from immune elimination (14). Therefore, monoclonal antibodies of anti-PD-1/PD-L1 have been developed to treat cancer and have achieved significant efficacy in the clinic therapy (15). However, some patients developed autoimmune diabetes after receiving antibody therapy (16). Thus, immunosuppressive checkpoint ligands on β-cells may play an important role in maintaining immune tolerance and protecting cells from the development of T1D (17). However, these immunosuppressive checkpoint ligands such as PD-L1 have been demonstrated as novel and potent therapeutic candidates to treat T1D. Additionally, Tregs can directly inhibit the activity of effector T cells. Therefore, Tregs play a critical role in peripheral immune tolerance and the prevention of autoimmune diseases (10). To restore the immune tolerance of the pancreatic microenvironment, it is essential to induce the expansion of Tregs (10). Autoantigen-based vaccines such as insulin and GAD 65 peptides could induce autoantigen-specific Tregs to treat T1D (18).

**Figure 1:**
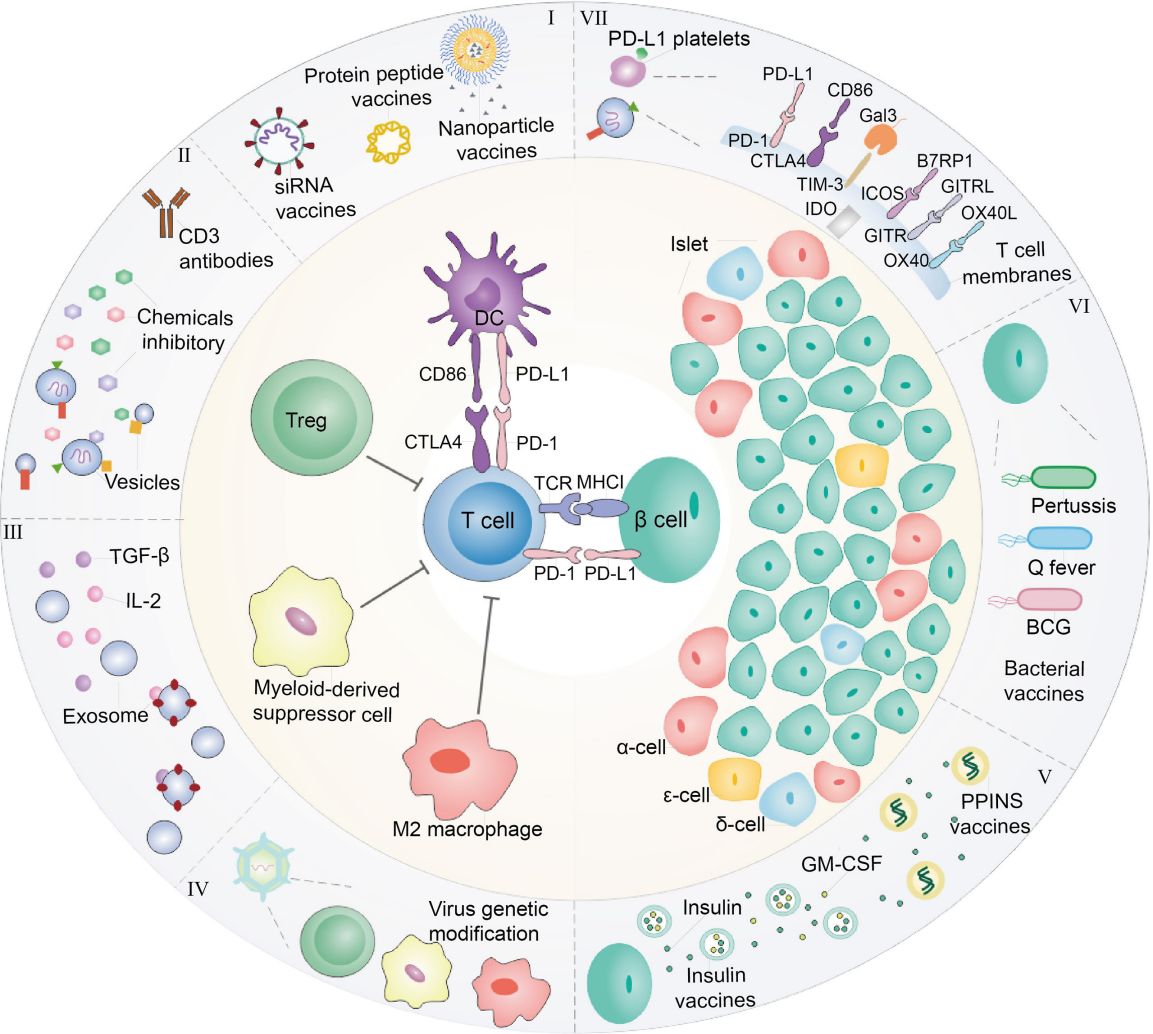
Diagram of interaction between immune cells and β-cells. At present, immunotherapy is commonly explored to treat T1D. (I) In the perspective of DCs, nanoparticles, or liposomes encapsulated with GAD56 peptides, siRNA molecules in pancreatic associated APCs Sema3E, Protein gene as T1D vaccines are often used. (II) For Tregs, the recovery of immune tolerance and immune homeostasis can be achieved by the use of CD3 monoclonal antibody and the combination with chemical inhibitors (prednisone azathioprine cyclosporin rapamycin et al.) to treat T1D. In order to enhance the therapeutic effect and reduce the toxic and side effects, it is considered to encapsulate the drug within nanoparticles or vesicles. (III) In addition, immunosuppressive factors such as transforming growth factor-β (TGF-β) and Interleukin-2 (IL-2) also have been employed for treating T1D. (IV) Immunosuppressive cells including Tregs, MDSC and M2 macrophage were cultured or engineered to modulate immunity of T cells for T1D therapy, which also could secrete exosomes with overexpression of TGF-β and IL-2. These immunosuppressive cells are often stimulated *in vivo* or re-infused into the body after the selective amplification or virus genetic modification *in vitro* to enhance their function of inhibiting T cells. (V) In addition, the insulin gene is modified and edited to produce insulin peptide vaccine, which can continuously secrete insulin in the body with only one injection. In cases where the β-cells have been damaged finally cause hyperglycemia, hence, foreign insulin is often utilized to maintain blood sugar levels. In addition to direct insulin injection, insulin and GM-CSF wrapped in cell membrane vesicles are currently considered to be injected into the body to achieve the purpose of sustained release. (VI) Moreover, β-cells are susceptible to bacterial infection and cause to damage. Therefore, it is common to detoxify pathogenic bacteria and make vaccines to achieve the purpose of prevention or treatment of T1D. Q fever vaccines, BCG vaccines and Coxsackie virus B1 vaccines are common bacteria that are used as T1D vaccines. (VII) On the other hand, T1D is caused by the direct β-cells attack by T cells. Thus, blocking T cells can be used to protect β-cells. For example, platelets expressing PD-L1 can accurately identify pathological T cells, and bind to PD-1 on T cell to abate its attack. Beside PD-1/PD-L1 immune checkpoint, CTLA4/CD86, TIM3/Gal3, ICOS/B7RP1, etc., also could be considered as T1D therapy targets.

Although the systemic immunosuppression could protect residual β-cells from autoimmune attack and reverse the diabetes, it still makes patients under the risk of organ damage. We discuss the recent advances in biomaterial used to immunomodulate immunity for T1D, including the controlled release of chemical immunosuppressive agent, targeted delivery of biological macromolecule for autoreactive T cell regulation by nanotechnology, exploring the biomaterials to develop diabetic vaccine and facilitating immunosuppressive cells to restore immune tolerance in pancreas (**Figure 1**).

## 2 Immune suppressive agents

As previously described, T1D is the result of deficiency of endogenous insulin due to the cell-mediated autoimmune destruction of pancreatic β-cells. Thus, how to preserve β-cells for sufficient production and release of insulin would provide a platform. To date, there have been several methods to enable the strategy of immune intervention: by either blocking autoimmune response against β-cells or achieving the replication of preexisting β-cells.

### 2.1 Prednisone

Prednisone is a synthetic glucocorticoid (GC) prodrug that is converted by the liver into prednisolone (a β-hydroxy group instead of the oxo group at position 11), which is a active steroid. In current study, prednisone is a synthetic corticosteroid, was administered to patients with T1D to roughly mimic the natural hormonal response to acute stress and to induce insulin resistance. Genomic effects are mediated by glucocorticoids, which bind to specific receptor proteins in target tissues and direct their production or inhibition. Therefore, most of the effects of glucocorticoids may not be seen for several hours.

Insulin requirements to maintain normoglycemia during glucocorticoid therapy and stress are often difficult to estimate. To simulate insulin resistance during stress, adults with T1D were given a three-day course of prednisone. Studies by Wendy C. Bevier et al. suggest that in adults with T1D, insulin requirements during prednisone-induced insulin resistance may need to increase by 70% or more to normalize blood sugar levels (19). Moreover, A. Secchi. et al. observed the curative effect of prednisone on the remission of newly diagnosed T1D patients and found that prednisone can also induce the remission of insulin dependent diabetes mellitus (IDDM). Remission may occur early in the onset of adult T1D (20). Prednisone broadly inhibits inflammatory cytokines caused by RTK bypass signaling and inhibition of EGFR or other RTK targets and thus may be a useful drug in combination with RTK pathway inhibition. Ke Gong et al. found similar results with dexamethasone, suggesting this could be GC. In particular, prednisone induces bypass RTK signaling, shutting down and repressing key resistance signals (21).While prednisone has a number of side effects and can induce significant problems with sustained use, it continues to be widely used in multiple inflammatory and rheumatologic conditions because it is highly effective.

### 2.2 Azathioprine

Azathioprine (AZA) and thiopurine are widely used for induction and maintenance of remission in steroid dependent patients with inflammatory bowel disease (IBD) (22). As the antilymphocyte adaptive agent, azathioprine and prednisone could induce immunosuppression and reduce insulin requirements at the onset of diabetes. However, the remission of autoimmunity was limited by the duration of the treatment, demonstrating that the tolerance to pancreatic β-cell autoantigens was not re-established (23).

Geliebter et al. delivered azathioprine after thymectomy for the treatment of T1D, which exhibited a good therapeutic effect on T1D. And Sun-275 azathioprine for slow release for the tertiary prevention of diabetes in newly diagnosed T1D patients, showing more effective results (24). Insulin dosage in patients with T1D can be effectively reduced by the delivery of Prednisone (19).

### 2.3 Cyclosporin A

Cyclosporine A (CsA) is an immunosuppressant agent that is widely used in clinical practice, predominantly for the prevention of rejection in various types of post-allogenic organ transplantation. In particular, cyclic peptides combine several properties such as high affinity, target selectivity and stability (enzymatic and chemical), which are important properties for therapeutics and make them ideal as orally delivered candidate drugs. The current interim analysis of the prospective and randomized reverse study showed that switching from tacrolimus to cyclosporine improved glucose metabolism and reversed new-onset diabetes after transplantation (NODAT) in majority of patients. The change of drug used was safe and did not result in an increased incidence of acute rejection episodes. Stiller C.R. et al. showed that the utilization of cyclosporine reduced insulin requirement and improved glycemic control in patients with recent T1D (25). However, cyclosporine could not fully establish immune tolerance and had other toxic side effects (25, 26). Furthermore, CsA induced nephrotoxicity, and azathioprine was ineffective as a single agent (23). Although some patients could achieve short-term control of diabetes, more researches are required to determine whether cyclosporine can be used safely to maintain glycemic control and prevent long-term consequences of the disease.

Short-term control of diabetes can be achieved by delivering Cyclosporin. Persistent remission appears to be dependent on continued administration of cyclosporine. Drug delivery decreases not only the leucocyte migration inhibition as previously observed (25), but also the lymphocyte-mediated cytotoxicity, which represents the late stage of cellular immune reactions against pancreatic tissue. Hence, it is promising to target the pancreas to control release of CsA *in vivo* through nanoparticles, such as cell membrane vesicles, polymer material PEG wrapping could achieve targeting therapy and reduce the toxicity of CsA for T1D.

### 2.4 Rapamycin

Rapamycin (RAPA) is a macrolide anti-immune antibiotic produced by Streptococcus. RAPA cannot directly block the transcription, translation and the secretion of gene products (IL-2, IL-3, IL-4, CM-CSF, TNF-α and IFN-γ, etc.) RAPA is different from nucleotide biosynthesis, it inhibits immune activation pathways that are insensitive to CsA and FK506. through blocking Ca2+-independent T cell activation and inhibiting IL-2-dependent entry of activated T cells into S phase.

Further, RAPA has been used in clinical transplantation for many years (27), including islet transplantation in T1D patients safely (28). In mice, RAPA monotherapy prevented from developing T1D but failed to induce disease reversal (29). Studies have shown that RAPA, as a targeted inhibitor of the mTOR pathway, exerts anti-inflammatory and anti-tumor effects in a variety of diseases by inhibiting the activation of the mToR pathway (30, 31). Interestingly, RAPA/GABA combination therapy effectively suppressed autoimmune responses to islet cells and improved islet function in patients with new-onset diabetes. In particular, after a hyperglycemic episode, patients treated with the RAPA/GABA combine significantly achieved diabetes improvement compared with patients treated with RAPA or GABA alone. This protective effect of RAPA/GABA combination treatment in NOD mice works through two distinct mechanisms (32). RAPA induces Tregs and thus suppresses targeted autoimmune responses to islet cells, which may be related to the reduction in insulitis observed in RAPA-treated NOD mice simultaneously. Manirarora and Wei treated NOD mice with a combination of IL-2/anti-IL-2 Ab complex, islet Ag peptide, and RAPA. Following combination treatment, CD4^+^CD25^+^Foxp3^+^ Tregs significantly expanded *in vivo*, expressed canonical Tregs markers, enhanced suppressive function *in vitro*, and spontaneously inhibited T1D in NOD mice. This novel approach to peripheral immune regulation will facilitate the progress of new immunotherapeutic strategies to prevent the development of T1D or promote resistance to islet transplantation without the long-term use of immunosuppressive drugs (33).

Lee Jung Seok et al. show that polymerized ursodeoxycholic acid, selected from a panel of bile acid polymers and formulated into nanoparticles for oral administration of RAPA, delayed diabetes in mice with chemically induced pancreatic inflammation attack (34). Nanoparticles acted as protective insulin carriers and high-affinity bile acid receptor agonists, increased intestinal absorption of insulin, polarized intestinal macrophages to an M2 phenotype, and preferentially accumulated in mouse pancreas, where they interacted with pancreatic islets associated with cellular bile. These nanoparticles could bind to acidic membrane receptor TGR5 with high affinity and activate glucagon-like peptide and endogenous insulin secretion. In mice, the nanoparticles also reversed inflammation, restored metabolic function and prolonged the animals’ survival. The metabolic and immunomodulatory functions of ingestible bile acid polymer nanocarriers may provide translational opportunities for the prevention and treatment of T1D. Moreover, RAPA can be modified. For example, drug delivery in cell membranes or cell vesicles, or in combination with other biological materials.

### 2.5 Anti-CD3 antibody

CD3 has five peptide chains, namely γ chain, δ chain, ϵ chain, ζ chain and η chain, all of which are transmembrane proteins. The transmembrane region of the CD3 molecule is connected to the transmembrane region of the two TCR peptide chains through a salt bridge to form a TCR-CD3 complex, which together participate in the recognition of antigens by T cells. The activation signal generated by TCR recognition of antigen is transduced into T cells by CD3. Preclinical studies suggested that mAbs against CD3 could reverse hyperglycemia at presentation and induce tolerance to recurrent disease. Based on previous study, the anti-CD3 monoclonal antibody could induce immune-suppressive Tregs and restoration of self-tolerance (35). Moreover, anti-CD3 specific antibody was used with a human proinsulin II to achieve remission even reversion of diabetes by synergistic effect (36). Interestingly, transgenic Lactococcus lactis combined with oral insulin and IL-10 combined with low-dose anti-CD3 in T1D mice can restore autoimmune tolerance and block the killing of β-cells by T cells (37). In addition, Stewart et al. combined a subcutaneously administered dual-size biodegradable microparticle (MP) platform with anti-CD3 to treated NOD mice. According to MP platform, DCs recruitment can be increased and Tregs expression can be induced to achieve immune tolerance. In theory, the combination of MP platform and anti-CD3 in the treatment of T1D mice seems to be a good direction. Sadly, there was no therapeutic benefit when considering the combination of biomaterials with CD3 antibodies (38). In this way, biomaterial combination therapy for T1D can be better utilized.

## 3 Immune suppressive factor

Immunosuppressive cytokines mainly include immune checkpoint molecules such as TGF-β, IL-2 and CD3. TGF-β is an important factor in inducing macrophages towards M2-type polarization and can promote IL-10 expression (39).IL-10 reduces the expression of MHC class II molecules and co-stimulatory molecules CD80 and CD86 on the surface of antigen presenting cells (APCs) such as dendritic cells (DCs) and macrophages, and mediates the phosphorylation of STAT3, and interferes with IFN-γ-induced monocyte activation and expression, leading to the function inhibition of APCs.

### 3.1 TGF-β

TGF-β inhibits effector cells directly or indirectly by disrupting DCs differentiation, migration, and antigen presentation. That is, TGF-β has two effects, both directly inhibiting T cells and inducing Tregs to establish a tolerance mechanism (40).

**Figure 2:**
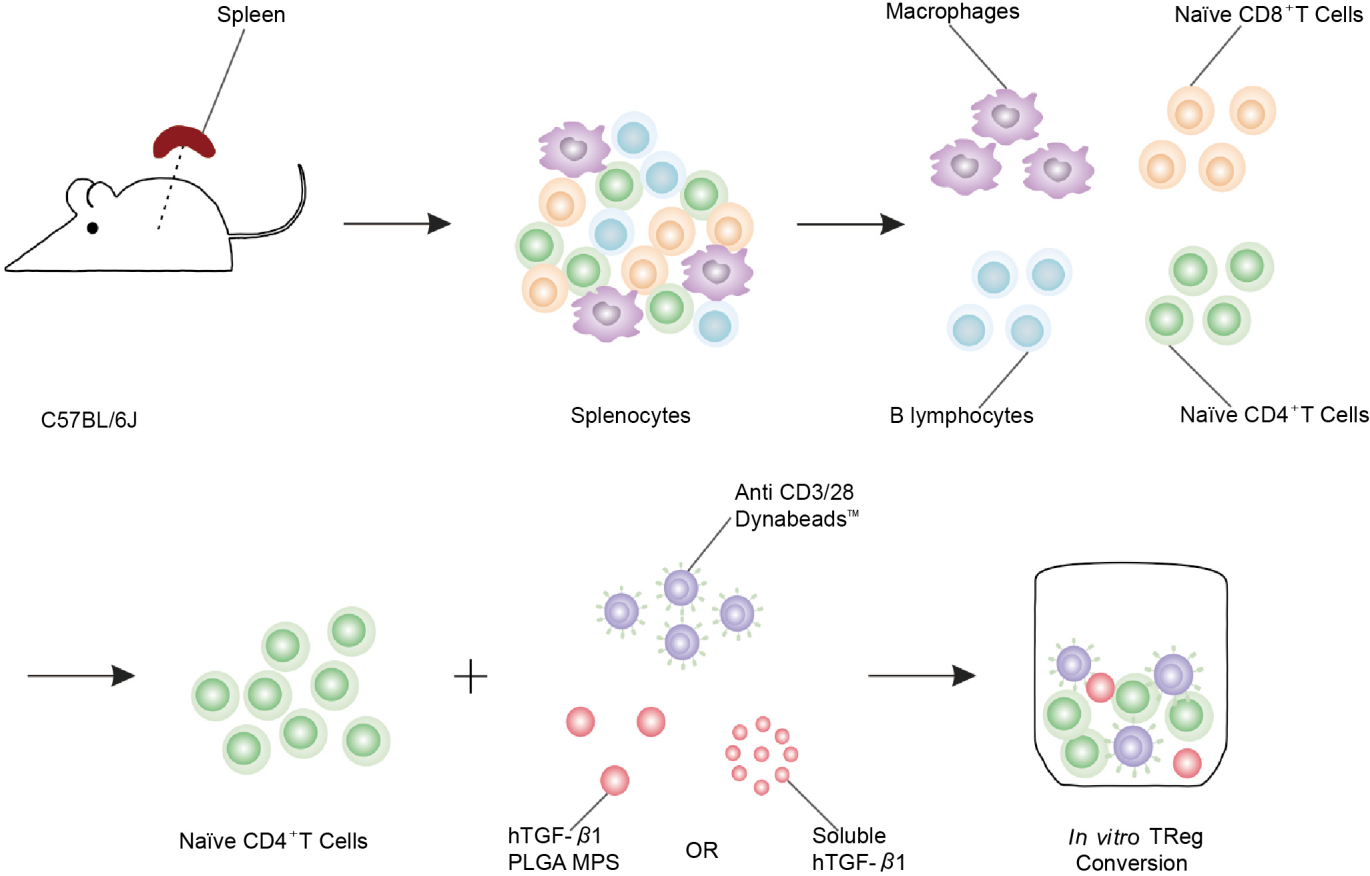
Schematic of polyclonal induced Treg (iTreg) conversion assay. Poly (lactic-co-glycolic acid) (PLGA) microparticles (MPs) were designed for the local and controlled release of immunomodulatory TGF-β1. PLGA MPs release TGF-β1, which can efficiently promote the conversion of naïve CD4+ T cells isolated from spleen to iTreg (41).

TGF-β is an immunoregulatory cytokine that enhances the development of an immune tolerance microenvironment by inhibiting antigen-specific inflammatory processes such as those found in autoimmune diseases and cancer. Li et al. used *in vitro* CD4^+^ T cell flow cytometry to verify forkhead transcription factor positive (Foxp3^+^) expression and TGF-β release (**Figure 2**) (41). In a TGF-β-rich environment, DCs, which uptake antigens, become tolerogenic and mediate the differentiation of antigen-specific CD4^+^ T cells into anti-inflammatory Tregs (42), which inhibits both the formation of antigen-specific effector CD4^+^ T cells and the activation and proliferation of cytotoxic CD8^+^ T cells (43). In order to avoid unavoidable inflammatory response induced by biomaterial implantation itself, TGF-β was designed to release from layered PLG scaffolds and showed great potential to enhance the function of encapsulated cells by decreasing the production of inflammatory cytokine (44). Considering immunomodulatory effect of MSC, in this study, researchers engineered MSCs with TGF-β gene to increase MSC potency for T1D therapy in mouse model. Engineered TGF-β/MSCs could restore several T1D functions, including modulating harmful immune responses, and could be a powerful tool for cell therapy of T1D compared to MSCs alone (45). To achieve sustained stimulation of regulatory immune cells, Ivan Koprivica et al. orally introduced microparticles (MPs) loaded with all-trans retinoic acid (ATRA) and transforming growth factor-β (TGF-β) to C57BL/6 mice treated with multiple low doses of streptozotocin (MLDS) for T1D induction. Mice treated with ATRA/TGF-β MP had significantly reduced disease incidence and immune cell infiltration into islets. In conclusion, oral administration of ATRA/TGF-β MPs ameliorated T1D by enhancing tolerogenic dendritic cells (tolDCs) and Tregs, suppressing Th1 responses, and preventing immune cells from entering islets (46). Current clinical practice relies on long-term systemic immunosuppression, leading to serious adverse events. To circumvent these adverse effects, polylactic-co-glycolic acid (PLGA) microparticles (MPs) were designed for localized and controlled release of immunomodulatory TGF-β1. *In vitro* co-incubation of TGF-β-releasing PLGA MPs with naive CD4^+^ T cells efficiently generated polyclonal and antigen-specific inducible regulatory T cells (iTregs) with potent immunosuppressive capabilities. Co-implantation of TGF-β1-releasing PLGA MPs with Balb/c mouse pancreatic islets into the extrahepatic epididymal fat pad (EFP) of diabetic C57BL/6J mice resulted in rapid allograft engraftment and local compatibility with PLGA MPs. TGF-β1 release is supported. However, the presence of TGF-β1-PLGA MPs did not confer significant graft protection compared to untreated controls, although insulin expression did not change and intra-islet CD3^+^ T cell infiltration was reduced and peripheral CD4^+^ T cells were measured elevated in - long-term graft site functional grafts. Examination of the broader effects of TGF-β1/PLGA MPs on the host immune system suggests a local nature of the immunomodulation, with no systemic effects observed. In conclusion, this approach established the feasibility of local and modular microparticle delivery systems for immunomodulation at liver explant sites. This approach can be readily adapted to deliver larger doses or other agents in the local graft microenvironment, similar to multi-drug approaches to prevent graft rejection (41).

Recent studies have demonstrated the unique potency of cell surface-bound TGF-β1 on Tregs to promote infection tolerance, i.e., confer suppressive capacity from one cell to another. To mimic this property, TGF-β1 was selectively chemically bound to an inert and viable polymer grafting platform using staudinger ligation. Inert beads tethered with TGF-β 1 were capable of efficiently converting naïve CD4^+^ CD62L^hi^ T cells to functional Treg. These results illustrated the unique function of the tethered TGF-β1 biomaterial platform to function as “infectious” Tregs and provide a compelling approach to generating a tolerant microenvironment for allogeneic transplantation.

### 3.2 IL-2

As IL-2/IL-2R might be the activation pathway relative to T1D (47), the safe and effective dose range of IL-2 for T1D therapy was investigated, showing that appropriate dosage of IL-2 facilitated β-cells persistence (47–49).What’s more, a recent clinical trial verified previous conclusion that IL-2 is a major role both in the development of T1D and the function of Tregs (50).

However, it is necessary to optimize the specificity of IL-2 since it can both activate Tregs cells and pathogenic T cells. Two major strategies have showed to be feasible in this area. One is antibody-cytokine conjugates. It was mentioned that different IL-2 and IL-2 monoclonal antibodies (mAbs) complexes could be used to achieve opposite effects. For example, IL-2 could enhance the proliferation of CD8^+^ T cells by binding to JES6-1 mAbs, as well as stimulating CD4^+^ Tregs with the combination of S4B6 mAbs, according to its selectivity resulting from conformational change (51, 52). And the other is chemical modifications of IL-2. A human IL-2, whose conformation was modified, could specifically expand Tregs *in vivo* to avoid off-target undesired effects (53). UFKA-20, an anti-human IL-2 antibody, was also identified and demonstrated the preference of stimulating CD4^+^ Tregs cells through binding to IL-2 (54). H9-RETR was designed as an engineered IL-2 variants and specific for IL-2Rb, holding the promise of a novel approach for treating T1D (55).

In addition, IL-2 conjugates with polymers, such as PEGylated IL-2, exert promising progress and are under further study. PEGylated IL-2 molecules that preferentially bind to different IL-2R conformations are being explored in oncology to controllably activate the IL-2 system and shift the balance in the tumor microenvironment in favor of effector T cells (Teffs). Overwijk et al. induced tumor regression by increasing intratumorally proliferation, activation and effector function of CD8^+^ T and NK cells (56). Cristina Izquierdo et al. demonstrated that combined treatment of Ag7/2.5 mi tetramer and IL-2: mAb complex is effective in preventing T1D. The treatment led to a large expansion of Ag-specific Foxp3^+^ Treg that acquire markers of activation, suppression and homing, and is accompanied by the proliferation of an antigen-specific Foxp3^−^ population that produced anti-inflammatory IL-10 (57). Jonathan T. Sockolosky et al. engineered IL-2 cytokine-receptor orthogonal (ortho) pairs that interact with one another, transmitting native IL-2 signals, but do not interact with their natural cytokine and receptor counterparts. Introduction of ortho IL-2Rb into T cells enabled the selective cellular targeting of orthoIL-2 to engineered CD4^+^ and CD8^+^ T cells *in vitro* and *in vivo*, with limited off-target effects and negligible toxicity. OrthoIL-2 pairs were efficacious in a preclinical mouse cancer model of adoptive cell therapy and may therefore represent a synthetic approach to achieving selective potentiation of engineered cells. OrthoIL-2 may be a synthetic approach to achieve selective enhancement in engineered cells (58). By modifying IL-2, the half-life of the drug can be prolonged, the cell specificity can be improved, and it can act more specifically on effector T cells or regulatory T cells for the treatment of tumors or autoimmune diseases. The sustained-release inhibitors used in the treatment of tumors can be used in the treatment of diabetes to improve the targeting of drugs.

## 4 Immune checkpoint

Immune checkpoints are immune homeostasis negative regulatory mechanisms that maintains autoimmune tolerance (59). Currently known immune checkpoints include programmed death-1/PD-L1(PD-1/PD-L1), cytotoxic T lymphocyte antigen 4 (CTLA4), Galactose lectin 3 (Gal3), indoleamine 2,3 dioxygenase (IDO) and so on. Of these, PD-1 and CTLA4 are the most well-known (60), although their modes of action and signaling pathways are distinct, they both contribute to maintain autoimmune tolerance. PD-1 is absent in immature T cells and memory T cells at rest, but it is expressed when the TCR is activated. Meanwhile, transcriptional activation is required for PD-1 expression on activated T cells. A classical immunoreceptor tyrosine inhibitory motif (ITIM) and an immunoreceptor tyrosine switch motif (ITSM) are also found in PD-1. On the other hand, CTLA4 is involved in down-regulating the magnitude of T cell response, mainly by competing with CD28 to share ligands CD80 (B 7.1) and CD86 (B 7.2) (59, 61). Nivolumab (PD-1 antibody), lambrolizumab (PD-1 antibody), and ipilimumab (CTLA4 antibody) are the most common immune checkpoint mAbs on the market today. These medications, rather than targeting tumor cells. As a result, immune checkpoint inhibition is frequently employed during tumor treatment to break tumor immunological tolerance. T1D, on the other hand, can move in the reverse direction of treating tumors, reverting over immunity to immunological homeostasis.

### 4.1 PD-1/PD-L1 targeting therapy

The immune checkpoint signaling axis PD-1/PD-L1 can effectively control T cell activity and prevent autoimmune attacks. To generate a self-amplifying signal loop, IFN-γ and IL-2 may increase PD-L1 expression, reconstruct the PD-1/PD-L1 inhibitory axis, suppressing CD4^+^ T cell activation and restoring immunological tolerance to over-activated T cells and over-produced pro-inflammatory cytokines (60, 61). It has been consistently proven that PD-1/PD-L1 interaction plays a central role in the induction and regulation of autoimmune diabetes progression in NOD mice (62). In addition, to gain more about how CD4^+^ T cells affect diabetes, Pauken transferred a small number of naive CD4^+^ T cells from BDC2.5 mice into prediabetic NOD mice, allowing the cells to be activated by endogenous autoantigens. The transferred BDC2.5 Tcells were activated and differentiated into T-BET ^+^ IFN-γ producing cells, which infiltrated the pancreas. The absence of PD-1 on CD4^+^ T cells leads to an increase in the number of cells in the spleen, pancreatic draining lymph nodes, and pancreas. At the same time, PD-1 deletion also increased the expression of chemokine receptor CXCR3. That is, PD-1 regulates islet responsive CD4^+^ T cells in a cellular manner by inhibiting proliferation, restraining pancreatic invasion and limiting diabetes mellitus (63). However, tumor patients developed insulin-dependent diabetes after taking the anti-PD-1 antibody (nivolumab) (64). Thus, the safety of systemic administration of anti-PD-1 antibody remains challenging.

**Figure 3:**
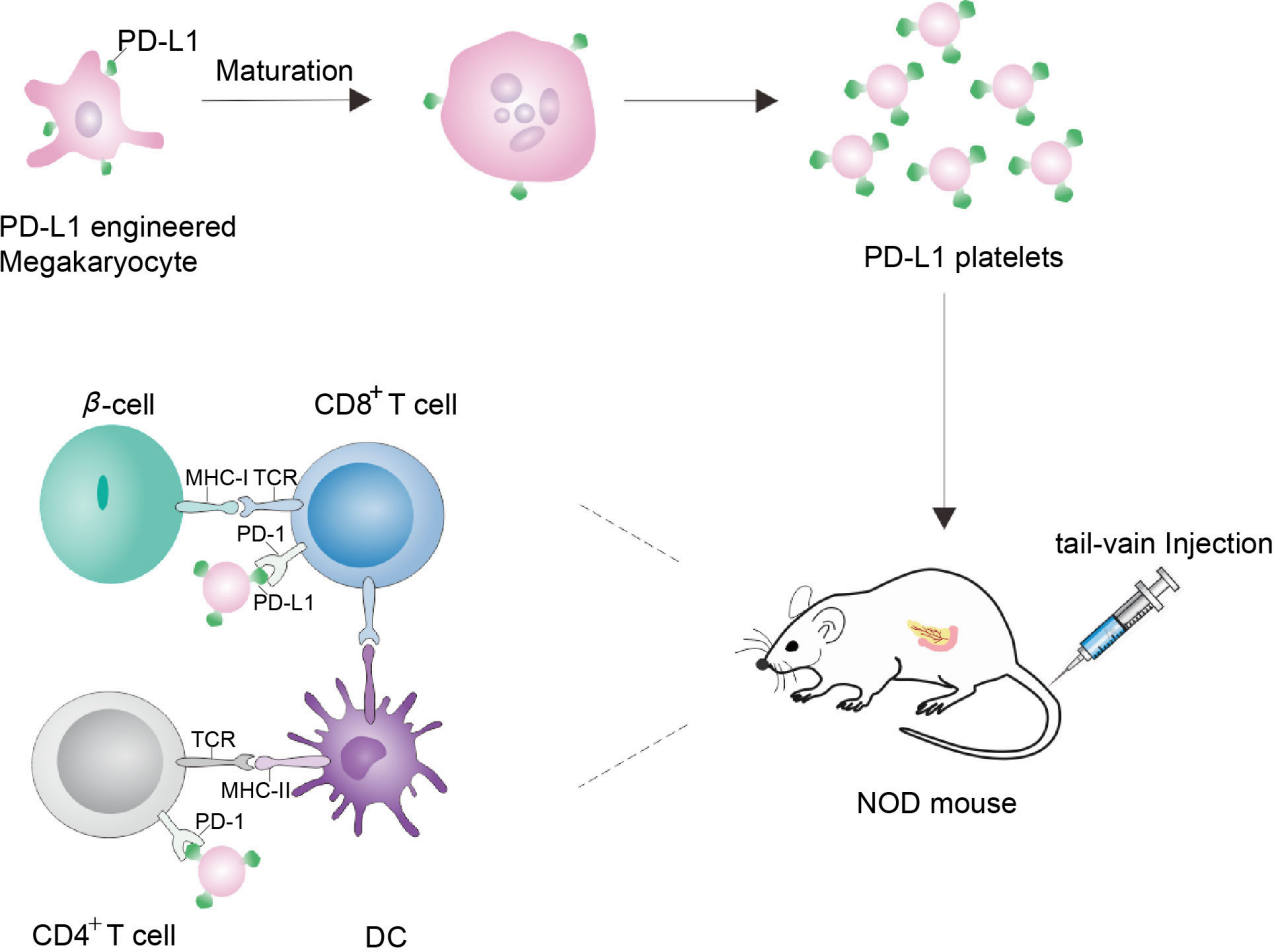
Schematic diagram of NOD mice treated by genetically engineering megakaryocytes to secrete platelets expressing PD-L1. Megakaryocytes were genetically engineered to express PD-L1. As PD-L1-megakaryocytes grow mature, the fragmentation of the proplatelets released platelets. Via tail-vein injection, PD-L1 platelets tend to accumulate in the inflamed pancreas, where PD-L1 can bind to PD-1 on the surface of CD4^+^/CD8^+^ T cells to protect β-cells, thereby protecting the pancreas and realizing the treatment of NOD mice (66).

In NOD mouse model, β-cells and hematopoietic stem and progenitor cell (HSPC) lack PD-L1, making them vulnerable to CD4^+^ T cell attack. Ben Naser et al. established a PD-L1 deficient HSPC mouse model to explore PD-L1 immunotherapy. They engineered mouse Lineage^−^c-kit^+^ (KL) cells *in vitro* to produce PD-L1 and transplanted the modified KL cells into a mouse pancreas. PD-L1 expression defects identified in T1D patients can be regulated by drugs encapsulated in human HSPC that could also inhibit *in vitro* autoimmune responses (65). Xudong Zhang et al. used genetically engineered megakaryocytes to overexpress PD-L1 and produced platelets with immunosuppressive function. In neo-hyperglycemic NOD mice, platelets overexpressing PD-L1 accumulated in the inflammatory pancreas, inhibited the activity of autoreactive T cells, protected insulin-producing β-cells from destruction, and maintained immune tolerance in the pancreas (**Figure 3**) (66). Yoshihara et al. generated human islet-like organs (HILOs) from induced pluripotent stem cells, which were driven by atypical WNT4 signaling for metabolic maturation. The cells were cultured into 3D multicellular spheres (MCSs) through gel, and the MCSs was coated with sodium alginate. It was transplanted into the renal sac of NOD/SCID mice with streptozotocin (STZ) induced diabetes. For allografts, they protected HILO xenografts with overexpression of PD-L1. The blood glucose of STZ mice returned to normal 50 days after allograft (67). Au et al. uses pretargeting and glycochemistry ways. The β-cells were first being delivered with nanoparticles encapsulated with targeted Ac_4_ManNAz, enabling β-cells to have high levels of surface-active azide groups. The PD-L1 immunoglobulin fusion protein (PD-L1-IG) was then given dibenzylcycloctane (DBCO) function. PD-L1 can easily bind to the surface of natural β-cells. In NOD mice, it was demonstrated that bioorthogonal staining promoted azide-alkyne cycloaddition effectively and selectively conjugated PD-L1 to β-cells. Moreover, *in vivo* functionalized β-cells present both islet specific antigen and PD-L1 to participating T cells, reversing early-onset T1D by reducing IFN-γ-expressing cytotoxic T cells and inducing antigen-specific tolerance (68).

However, immune checkpoint inhibitors also have adverse reactions in the treatment of insulin-dependent diabetes. Through 6 years of cases in two academic institutions, Stamatouli et al. found that a minority number of patients who received anti-PD-1 or anti-PD-L1 checkpoint inhibited developed ketoacidosis, pancreatitis and other autoimmune diseases (69). As a result, a new requirement for the development of PD-L1 for the treatment of diabetes is how to prevent the development of autoimmune diseases in a minority percentage of individuals.

### 4.2 Engineering CTLA4 for targeting therapy

CTLA4 is a T cell negative regulator that regulates T cell activation by competing with the costimulatory protein CD28 and binding to the shared ligands CD80 (B7.1) and CD86 (B7.2) (70). Meanwhile, the long-term survival of islet allografts produced by the soluble fusion protein CTLA4-immunoglobulin (CTLA4-Ig) is dependent on the host’s effective tryptophan catabolism. CTLA4 function *in vivo* by regulating tryptophan catabolism, but it also acts as a ligand of B7 receptor molecules, transducing intracellular signals (71). In order to treat diabetes mellitus, El Khatib et al. overexpressed synthetic PD-L1-CTLA4-Ig polyprotein using a cell-targeted AAV8 vector. Sadly, in NOD mice injected with proteins during early hyperglycemia, PD-L1-CTLA4-Ig alone was unable to normalize the state. However, the PD-L1-CTLA4-Ig expression vector prevented the islets from rejection for at least 4 months when drug-induced diabetic mice received MHC-matched transplanted islets. While transplanting PD-L1-CTLA4-Ig-expressing MHC-matched islets into T1D animals with established disease prevented immediate excretion of the transplanted islets, it eventually failed to sustain long-term immunological tolerance. Therefore, it is useful to design PD-L1 and CTLA4 gene-based strategies for the treatment of diabetes (72). Intriopirocrine green (ICG) is a photothermal agent to encapsulate imiquimod (Toll-like receptor 7 agonist) with polylactic acid coglycololic acid (PLGA). The formed PLGA-ICG-R837 nanoparticles, which trigger photothermal ablation by a near-infrared laser, can produce tumor-associated antigens *in vivo*, while combined with CTLA4 antibody therapy, which can prevent recurrence after tumor elimination. Can similar biomaterials be used for the treatment of T1D? In addition to the current treatment for diabetes with CTLA4-Ig, utilizing CTLA4 to prevent the onslaught of autoimmune cells to the islets is a suitable place to start (73). In reality, a “CAR-T” that expresses CTLA4-Ig was created in a method akin to CAR-T to continuous production of CTLA4-Ig *in vivo*, which can lessen the long-term immunosuppressive effectiveness and autoimmune rejection brought by islet transplantation.

### 4.3 Galactose lectin

Galactose lectin family plays an important role in cell proliferation and activation, but its function is easily reversed by the influence of the microenvironment. Galactose lectin has been continuously explored since 1975 when abnormal expression of galactose lectin was found in cancer. At present, Gal1 and Gal3 have been identified as the main molecules that mainly promote the growth of tumor cells and evade immune surveillance. At the same time, studies have suggested that the overexpression of Gal1 is related to the poor prognosis of patients with tumors, and the cure of hepatocellular carcinoma is often based on the expression of Gal1 (74, 75). Overexpression of Gal1 enables DCs to acquire interleukin-27 (IL-27) dependent regulation, promoting IL-10-mediated T cell tolerance and inhibiting autoimmune inflammation (76). It has also been found that Gal1 is related to the activation of MAPK signaling pathway and phosphatidylinositol-3 kinase (PI3K) signaling pathway. Gal9 in the galactose lectin family has shown great potential in the treatment of various cancers, and it was discovered that TIM-3 is a set of signaling pathways with Gal9 (77). The TIM-3/Gal9 pathway does not play a direct immunosuppressive role. Instead, TIM-3^+^ T cells were used to interact with CD4^+^CD25^+^ Tregs expressing Gal9 to activate T help 1 response and achieve immunosuppression (78).

In addition, Gal3 can also control the release of inflammatory cytokines into the body and the uptake of glucose by adipose tissue. Among some of the lectins, Gal3 is mostly secreted by macrophages. When fed a typical diet, without any accompanying inflammatory disease, galactose agglutinin-3 deficient (LGALS3 ^-/-^) mice had smaller bodies and less epididymal white adipose tissue (eWAT). Further, metformin therapy could decrease these galactoagglutinin-3 levels (79). Mice receiving Gal3 injections developed insulin resistance and glucose intolerance, but obese mice receiving Gal3 treatment had enhanced insulin sensitivity due to pharmacological or gene loss. Gal3 was demonstrated to impair the insulin-mediated regulation of glucose output in primary mouse hepatocytes, lower the insulin-stimulated glucose uptake in muscle cells and 3T3-L1 adipocytes, and directly increase the chemotaxis of macrophages in *in vitro* tests. Gal3 can bind directly to the insulin receptor (IR) and suppress the following signals. So Gal3 inhibition is a novel target for the treatment of insulin sensitivity (80). Can pancreatitis be decreased or perhaps reversed by reducing or removing Gal3 since it is recognized to be the primary cause of pancreatitis? Iacobini et al. fed both wild-type (LGALS3^+/+^) and galactin-3 defective (LGALS3^-/-^) mice an atherogenic diet more than a longer length of time before the levels of fibrosis, inflammation, and steatosis in wild-type mice were much lower than those in wild-type mice. Gal3 was also discovered to be the receptor for advanced lipid oxidation end products in the liver. Gal3 ablation protected against diet-induced nonalcoholic steatohepatitis and reduced inflammation, hepatocyte damage, and fibrosis in terms of reducing the formation of lipoxidation end products in the liver (81). The application of the galactose lectin family is very promising, which can be considered as combined with other immunosuppressive agents, immunosuppressive factors, etc., to detreat T1D.

### 4.4 IDO

Initially, it was thought that the indoleamine 2,3 dioxygenase (IDO) enzyme directly affected how the immune system responded to illnesses and infectious agents. But as the years progressed, our knowledge of IDO has grown more and more comprehensive. In addition to limiting microbial growth and increasing immune response as an antibacterial, IDO may also have a role in modulating immune response activation and the development of immunological tolerance (82). The upregulation of plasma nephropathy (Kyn) by indoxyamide2,3-dioxygenase 1 (IDO1) in adipocytes has also been discovered. Mice were protected from obesity by IDO1 consumption in fat cells, which diminished Kyn buildup. For diabetic, obese mice, this offers a fresh perspective (83). Additional research on the location and expression of IDO in pancreatic tissues showed that diabetes patients with new-onset or other autoimmune diseases had significantly lower levels of IDO expression in insulin-secreting islets (84).

In C57BL/6 mice deficient in the Toll-like receptors9 (TLR9) gene, the lack of IDO induction in the pancreatic lymph nodes was likewise associated with the lack of STZ induction. It caused STZ mice to display some symptoms of a more serious illness. In order to prevent and treat T1D, attention should be paid to the TLR9-IDO axis (85). Due to decreased expression and catalytic activity of IDO1 in plasmacytoid dendritic cells (pDCs), TGF-β is unable to activate the IDO1 signaling pathway in NOD mice. Pallotta et al. pretransfected NOD pDCs with IDO1 to activate the IDO1 promoter, boost atypical NF-κB and TGF-β, and inhibit the production of pro-inflammatory cytokines, interleukin-6 (IL-6) and tumor necrosis factor-α (TNF-α), in such autoimmune diabetic animals. The presentation of pancreatic cell self-antigens *in vivo* could be inhibited by pDCs after TGF-β was applied to NOD pDCs with IDO1 overexpression (86). Moreover, the proteasome inhibitor bortezomib (BTZ) was utilized to restore IDO1 protein expression *in vitro* in pDCs. In autoimmune diabetic mice with prediabetes, treatment with BTZ can reverse the disease. But in hyperglycemic mice, there was no therapeutic efficacy (87). Dermal fibroblasts may also be used in addition to pDCs. dermal fibroblasts expressing IDO were given to newly unwell NOD mice by Zhang et al. in different doses. Only a small percentage of mice receiving low dosage therapy had their blood sugar levels reversed, but most animals receiving high dose treatment with dermal fibroblasts had their blood sugar levels returned to normal. It is amusing to note that high doses of IDO produced by fibroblasts reduced immune cell infiltration significantly while restoring islet cell function in NOD mice. Additionally, it lowered CD8^+^ T cells and T helper cell 17 in interactive NOD mice and increased Tregs in a number of organs (88).

There are different methods to accomplish IDO expression besides cell regulation. In inflammatory bodies, Dolpady et al. noticed that probiotics VSL#3, which were rich in lactobacilli, raised the release of IDO and IL-33 while actually lowering the expression of IL-1. By promoting CD103^+^ DCs differentiation tolerance and lessening Th1 and Th17 cells differentiation/expansion in intestinal mucosa and autoimmune disease, oral administration of lactobacteria-rich probiotics VSL#3 to NOD mice, either alone or in combination with retinoic acid, can modify their own intestinal microflora (89). Therefore, it makes sense to control IDO expression in a variety of methods in purpose of treating T1D.

## 5 Immunosuppressive cell therapy

As an autoimmune disease, T1D is induced by immune dysregulation, subsequently leading to the attack of pathogenic T cells to autologous β-cells. In view of this, several immunomodulatory therapies, such as delivering immunosuppressive agents and immune inhibitory biomacromolecule, have been proposed as described above. Most of them are focus on Treg, an immunosuppressive cell contributing to suppress overly pathogenic T cells and restoring autoimmune tolerance. However, more effective treatments are desired for complete remission of T1D due to the limited immune tolerance of the above therapies. Herein, adoptive suppressive immune cell therapy would be introduced to provide a broad overview of overcoming the above challenges.

### 5.1 Treg

**Figure 4:**
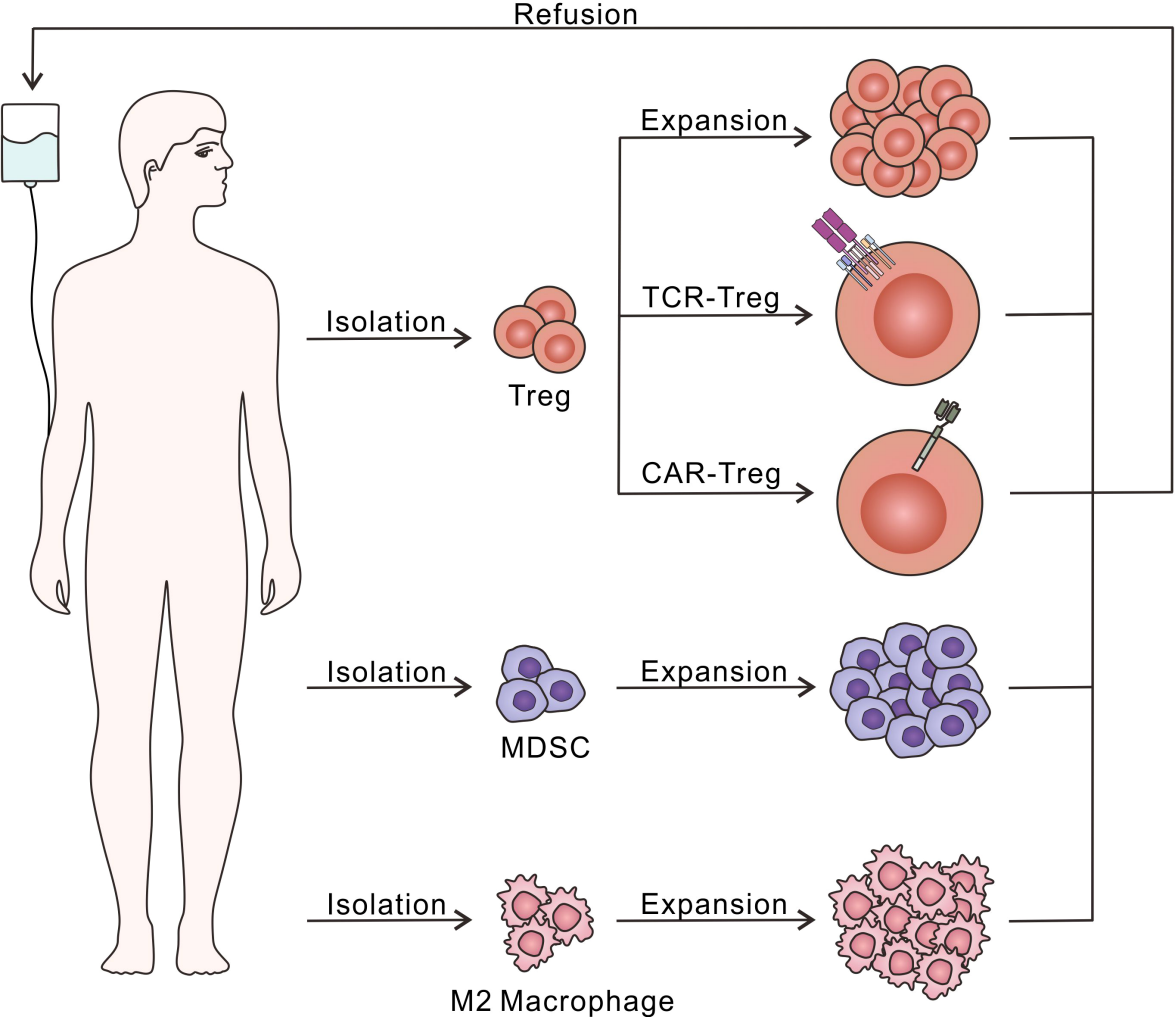
Diagram of adoptive immunosuppressive cell therapy. Treg, MDSC and M2 macrophage could be isolated and expand *in vitro* through different culture conditions, followed by the refusion into the host to relieve excessive autoimmune response. Moreover, Treg have been further modified with TCR or CAR to recognize T1D-related autoantigens, which is beneficial to enhance the immunosuppressive effects.

In a research based on mice, CD4^+^CD25^+^ Treg was found to play an important role in relieving immune response and preventing the process of various autoimmune diseases (90). The Foxp3 was then identified as a special marker of CD4^+^CD25^+^ Treg, regulating their development and function (91). How Treg exerts immunosuppressive effects was discussed (92), and the further mechanism is still under study. Herein we demonstrate Treg as a kind of cell-based biomaterial applied in T1D therapy (**Figure 4**).

#### 5.1.1 Expansion of Treg *in vitro*

Since natural CD4^+^CD25^+^ T cells generally keeps a small amount *in vivo*, the expansion *ex vivo* is considerable. In a research of Tang et al., decreased CD25 expression of Treg was observed in the islets of T1D mice. Moreover, low-dose IL-2 was found to be conducive to the survival of Treg and remission of T1D (48). Later, TGF-β/TGF-β receptor signals were demonstrated to have key roles in CD4^+^CD25^+^ Treg regulation (42). Therefore, IL-2 and TGF-β were used to co-cultured with naïve T cells, which would be stimulated to differentiate into CD4^+^CD25^+^ T cells (93, 94). Other *in vitro* factors, such as anti-CD3 and anti-CD28, acted as a way of indirect antigen presentation, were combined with fluorescence-activated cell sorting technology for better therapy (95). As for direct antigen presentation, DCs, function as APCs, were employed to expand Treg for stronger alloantigen-specific capacity, benefiting to ease autoimmune disease *in vivo* (96–98). With the low-dose antigen, DCs could stimulate CD4^+^ T cells to generate a group of antigen-specific Treg, and without additional purification, it could ameliorate T1D.

However, the marker of CD4, CD25, and Foxp3 could not define the whole Treg subset, since some of CD4^+^CD25^-^CD127^-^ T cells also express Foxp3. Further research demonstrated that CD127 could be an excellent Treg marker, especially for those suppressor cells without antigen presentation (99). CD4^+^CD25^+high^CD127^-^ Treg have been initially confirmed the prolonged survival of β-cells, insulin dependence and safety within one year of single or double Treg infusion (100)CD4^+^CD25^+^CD127^lo/-^ polyclonal Treg was isolated from patients, expanded *in vitro*, showing its enhanced suppressive activity *in vivo* after being re-infused in a phase 1 trial (101). *Ex vivo* expanded autologous CD3^+^CD4^+^CD^25high^CD127^−^ Treg was infused and contributed to the remission of T1D in children (102).

In some researches, stem cell-derived autoantigen-specific Treg was also applied in T1D treatment, showing autoantigen-specific and inhibition autoantigen-specific accumulation (103). A novel idea was validated to make Treg as a protective coating of transplanted pancreatic islets. Specifically, Treg was bound to target cells through streptavidin-biotin system or biotin-polyethylene glycol-succinimidyl valeric acid ester molecule, maintaining the activity and function of islets and preventing them from being immune attack (104, 105). However, *in vivo* experiments were required to further demonstrate the coating strategy.

Despite extensive research effort, it seemed that there were some of antigen-specific Treg playing a significant role to reverse T1D in the large number of *in vitro* expanded Treg, though the former represents only a very small fraction of the latter. In a T-cell exhausted mice T1D model, autoantigen-specific Treg showed superior capacity in T1D remission than polyclonal Tregs (106). Therefore, compared to selectively isolate and expand antigen-specific Treg, it may be a more effective pathway to engineer T cells to be antigen-specific Treg.

#### 5.1.2 Engineered Treg

As for the uncertainty of *in vitro* amplification, direct gene-edited Treg contributed an attractive route. T cell receptor (TCR), which recognizes T1D-related autoantigens, was constructed on Treg *via* lentiviral gene transfer and enabled its feasibility on Treg. Specifically, Treg was endowed with ectopic expression of TCRs, which was identified and isolated from T cells of human origin, leading to antigen dependent inhibitory capacity and protecting islets from β-cell destruction (107, 108).

Compared to TCR-Treg, Chimeric antigen receptors (CARs)-Treg owns a unique advantage of being non major histocompatibility complex (MHC)-restricted, which means CAR transfer would be applicable to most patients, instead of varying from individuals.

HLA-A2 specific CAR Treg maybe the first concept of proof for therapy. Herein, extracellular single-chain Fragment Variable (scFv) was fused to extracellular signaling domain, enhancing our insight into creating Treg of various antigen specificity *via* using different CARs (109). A modular system based on CAR-Treg was developed. Usually, CAR was designed to interact with specific target antigen. Herein, the CAR was engineered to bind to Fluorescein Isothiocyanate (FITC), a fluorophore frequently conjugated to antibodies, which endowed it with enormous scalability to act with desired antigen in prolonging allograft survival and maintaining immune tolerance (110). To increase Treg yield and further ensure its security, Foxp3 was transduced to CD4^+^ T cell to generate converted Treg (cTreg), exhibiting the similar phenotype and function as natural Treg. Moreover, insulin-specific CAR construction of cTreg could make it activated and proliferative. It fulfilled a concept-on-proof of redirecting T effector cells to Treg through transduce Foxp3 (111).

Despite promising results, a number of issues remain to be solved, especially in the long-term safety and validity, no matter the large-scale expansion technology, or gene-editing method like TCR Treg and CAR Treg, the lineage stability of Treg in manual operation remains unknown. Recent data indicated that no Foxp3 loss in expression during over a year follow-up period (101). However, to prove the inevitability between lineage stability and Foxp3^+^ of Treg, more robust evidence is needed. As for gene-editing, it is particularly worrying about Good Manufacturing Practice, including the construction and optimization of TCR/CAR, purification of TCR/CAR Treg. Considering the widely existing neurotoxicity CAR-T used in oncology research, CAR Treg may also face the similar challenging hurdles.

### 5.2 MDSC

A population cell of myeloid origin with immunoregulatory activity named myeloid-derived suppressor cells (MDSC) is of interest, allowing for great potency in the fields of transplantation and autoimmune diseases (112). In T1D, MDSC exhibited immunosuppressive ability to some extent, its characteristic and mechanism had been discussed in the pre-existing review (113). Here, we focus on the studies related to MDSC adoptive therapy.

Alessia Zoso et al. proposed a novel human MDSC subset named fibrocytic MDSC to induce Treg expansion and normoglycemia in a xenogeneic mouse model of T1D (114). However, there was another research pointed that the adoptive transfer of MDSC could not always effectively prevent T1D autoimmunity in a stringent model (115). Combined therapy with adjunct immunosuppressive agents or addition factors would be needed (115, 116). Whitfield-Larry F et al. utilized hemagglutinin (HA) peptides to assist MDSC, making it capable of downregulating autoimmune responses and preventing diabetes onset (116).

In addition to direct action in assisting the establishment of immune tolerance, MDSC also protected transplanted islets from being disrupted. It reported single dose of MDSC was given to the treated mice and prolonged the survival of transplanted islets, indicating MDSC owned the capacity of immunosuppression. Moreover, the expression of C-C chemokine receptor type 2 (CCR2) contributed to MDSC migration, which means much more supervision would be required (117).

However, one obvious drawback limits the development of MDSC, that is the small number of them within the whole body. It usually means that it would be difficult to precisely isolate the “appropriate” MDSC, especially in autologous patients. Generic MDSC from healthy donors may suffer from mismatch genetic background and further Graft Versus Host Disease (GvHD). Exploring extra *in vitro* inducers for better MDSC production and *in vivo* combinations for effective function has still the long way to go.

### 5.3 M2 macrophages

It has been reported that early macrophage infiltration plays an important role in the process of T1D (118). There were two major activation states of macrophage, including proinflammatory (M1) and anti-inflammatory (M2, also named alternatively activated macrophages). A strategy of alternatively activated macrophages was proposed and generated keen interest. Similar to adoptive transfer therapy with other cells, M2 macrophages was isolated from macrophages, monocytes, or bone marrow–derived myeloid precursors, followed by *in vitro* induction using cytokine such as IL-4 and colony-stimulating factor-1 (CSF-1) (119, 120). The mechanism of M2 macrophage protection has been illustrated in other review (121). Here we focus on the application of M2 macrophage therapy.

In STZ induced diabetic mice, adoptive transfer of M2 macrophage expanding *in vitro* showed mitigated injury of islets and kidneys, mainly due to the protection of M2 macrophage from the destruction of β-cells (120, 122). Roham Parsa et al. developed an optimized *in vitro* culture protocol, that was IL-4/IL-10/TGF-β. Most of NOD mice exhibited good therapeutic effects of T1D for more than three months after being injected the induced M2 macrophage. Surprisingly, it was observed that the transferred M2 macrophage was specifically accumulated in inflamed pancreas and promoted β-cell survival (123). A study revealed that M2 phenotypical cell line could be stabilized by neutrophil gelatinase‐associated lipocalin transduction, which overexpressed IL-10 and low secreted TGF‐β, relieving diabetic kidney disease much better than non-modified bone marrow‐derived M2 (124). A small-scale clinical trial indicated that monocyte of newly diagnosed T1D patients could differentiate M2 macrophages *in vitro* while that of long-standing T1D patients could not. The differentiated M2 macrophages could use for adoptive therapy (125). In our opinion, M2 plasticity make it a potent candidate for cell therapy through genome editing and reprogramming. However, the limited proliferation halted the progress, indicating more advanced co-cultured and modified methods are still needed to developed.

## 6 Vaccine

There is a lot of data to prove the point that viruses can induce T1D in animals. The mouse encephalitis myocarditis (EMC-D) virus D variant is the most prevalent. Due mostly to the rapid death of β-cells by virus replication in cells, high titer EMC-D virus infection progresses to diabetes within 3 days. Macrophages are drawn to the islets by low titer EMC-D virus infection. An important element in the elimination of remaining β-cells is the production of soluble mediators by activated macrophages (126–128). In addition to the above mentioned, autoimmune responses can be modified from immune checkpoints. It can also be done by antigen-specific pathways. In addition to the above mentioned, autoimmune responses can be modified from immune checkpoints. It can also be done by antigen-specific pathways. Vaccines have been suggested as a means of treating and preventing T1D at beginning with its development. By controlling the autoantigen immune response and halting further degeneration of pancreatic β-cells, vaccines have been demonstrated clear advantages in immunotherapy. It can not only significantly lessen the pain brought on by diabetes complications but even help reverse diabetes (18). Inflammation is a major contributor to T1D, hence, strategies for reducing or eliminating inflammation are frequently used in the vaccine selection and formulation process.

### 6.1 Bacteria

From the first pertussis vaccine found to stimulate the production of immunoglobulin by lymphocytes in rats to the later treatment of gas-sensitive inflammation in mice (129–131). In 1984, to reduce inflammation, a study explored the protective effect of pertussigen on insulin-dependent diabetic mice. The pertussis vaccine was divided into two parts, one was boiled and the other was left intact, and injected into STZ mice. T1D was not observed in mice treated with either regimen (132). It has been suggested to use Tregs to inhibit or remove pathogenic T cells in order to prevent or safeguard the function of β-cells. Trials have started in children with new-onset diabetes and adults with autoimmune diabetes (LADA) based on this notion (133). Traditionally Q fever vaccines have traditionally been used to treat acute febrile illness after human contact with livestock. After professor Kevin Lafferty first demonstrated that Q fever vaccine protects NOD mice against diabetes, Bonn et al. explored whether Q fever vaccine could prevent diabetes in humans. They vaccinated more than 40 newly diagnosed T1D patients with Q fever, patients did not develop a second autoimmune disease from vaccination to 6 months later. These results, making vaccine against hepatitis virus colonies be a new way to protect insulin-producing β-cells (134). On the other hand, the soluble chimeric protein CTLA4-Ig can treat psoriasis when injected into the body. No T cell proliferation was observed in the patient’s lesions, but there were abnormal antibody responses to T cell-dependent neoantigens (135). Bacille Calmette-Guerin (BCG), a derivative of Mycobacterium bovis, induces upregulation of the c-Mcy genes that controls glucose homeostasis and is most commonly used to trigger long-term adjustment of blood sugar levels to near-normal levels in patients with T1D (136, 137). In addition, patients with long-term T1D received two doses of BCG for 8 years of follow-up. The BCG vaccine returned blood sugar levels to normal for three years after administration and remained stable for the next five years (138). Therefore, it is promising to employ BCG to treat T1D. From the perspective of adjuvant, peptide nanofibers have been used to assist BCG vaccine expansion of CD4^+^ T cells (137). It may be possible to extract microvesicles of Mycobacterium bovis and load them with immunosuppressive drugs as a strategy to treat T1D. Rotavirus mainly infects intestinal cells and causes dehydrated gastroenteritis (139). Hence, children must be given rotavirus at an early age. Notably, between 2006 and 2017, the incidence of diabetes in children from aged 0 to 4 declined by 3.4% per year after infants were vaccinated against rotavirus in the United States (140). Rotavirus spike protein is considered as a novel vehicle protein, which can greatly improve antigen immunogenicity when combined with vaccine (36, 141). Thus, whether combination of rotavirus spike protein or MEC-D to make a preventive vaccine could prevent T1D caused by MEC-D.

Moreover, Miller et al. synthesized an oral live attenuated salmonella vaccine that delivers its own antigens and TGF-β expression vectors to immune cells in the intestinal mucosa. DCs are present throughout the secondary lymphoid tissue after animal vaccination, and CD103^+^ DCs induce tolerance effects and intestinal homing. TGF-β significantly increased the expression of programmed death ligand-1 (PD-L-1 or CD274) in dendritic cells in MLN and PP of treated mice. TGF-β also increased the level of CTLA-4 in CD4^+^ T cells in MLN and PP (142). Based on this, Harrison et al. made a T1D vaccines using live attenuated Salmonella MvP728 (DhtrA/DpurD), cytokines (IL10 and TGF-β) and proinsulin (PPI) antigen combined with a subtherapeutic dose of anti-CD3 monoclonal antibody. The vaccine was able to reduce insulitis and prevent and reverse diabetes in NOD mice. It also reduced the risk of inflammatory response and sepsis by detoxifying lipopolysaccharide (LPS) with lipid A against live salmonella strains (143). Considering that the vaccine itself may trigger autoimmunity and the selection of the enterovirus serotype that the vaccine targets, Larsson et al. used formalin inactivated Coxsackie virus B1 (CVB1) as a novel non-adjuvant vaccine to explore its efficacy and autoimmune safety in the treatment of diabetes. Prediabetic NOD mice were inoculated with CVB1 vaccine and then infected with CVB1. The vaccinated mice produced high titers of CVB1 neutralizing antibodies, but showed no sign of vaccine-related side effects and no increase in insulin autoantibodies. At the same time, the onset of diabetes was accelerated in NOD pre-diabetic mice infected with CVB1 (144). Bacterial vaccines have great potential for the treatment of diabetes, either by using bacterial proteins as adjuvants to enhance antigen immunogenicity or by combining nanoparticles with bacteria.

### 6.2 Genetic engineering

**Figure 5:**
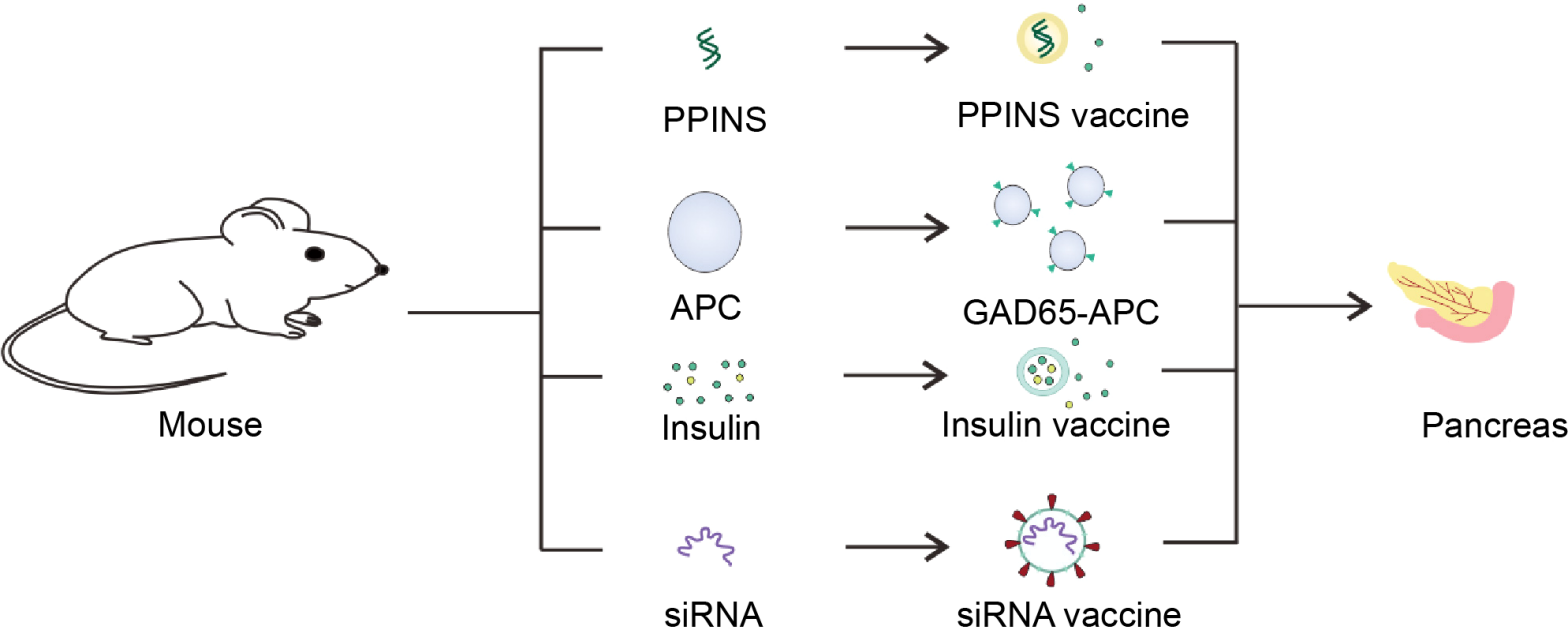
Schematic diagram of the T1D vaccine. Insulin gene was isolated from mice and utilized to made PPINS vaccine. Liposomes and other nanomaterials are used to coat insulin and GM-CSF to provide exogenous insulin, which are mainly used to make sustained release insulin vaccines. On the other hand, siRNA vaccines derived from siRNA molecules of APC associated with the pancreas and cell vaccines genetically engineered to express GAD65 antigen could protect the pancreas from T cell killing.

Genetic engineering has been a hot topic in recent decades. It is possible to use gene editing and other methods to give organisms functions that they do not have (**Figure 5**). For patients with T1D, it is of great significance to re-enshrine the function of producing and secreting insulin.

#### 6.2.1 Proinsulin

For patients with T1D, insulin injections are commonly used. However, considering long-term insulin injection, it will cause body edema, hypoglycemia and other adverse reactions. There are many ways to get insulin into the body. Apart from the common intraperitoneal injection, there are nasal inhalation, oral and so on. Under continuous stimulation of high glucose, insulin nasal mucosal injection can maintain the stability of blood glucose and autoimmune tolerance (143). Meanwhile, blood glucose levels can be reduced with oral insulin, however the first-pass effect can lower the quantity of insulin that is actually useful. Therefore, there are new difficulties in increasing the amount of insulin that is effective (145).

For daily insulin injections, a way to produce a continuous supply of insulin in the body with a single injection is urgently needed. Sifter et al. analyzed the structural characteristics of the variation in the proinsulin proteins (PPINS) and designed PPINS as antigenic subcellular targeted vaccines. The potential of PPINS variants to inhibit the development of spontaneous diabetes was then tested in female NOD mice expressing h-2G7 haplotype susceptible to diabetes with CD8^+^ T cell-reactive antigens. They found that PPINS antigens excluded from endoplasmic reticulum (ER) expression did not induce CD8^+^ T cells or autoimmune diabetes in RIP-B7.1 TG mice, but effectively inhibited the development of spontaneous diabetes in NOD mice and CD8^+^ T cell-mediated autoimmune diabetes mice (146). Glinka et al. co-inoculated PPINS with mutated B7-1 molecule (B7-1WA) to protect NOD mice prone to autoimmune diabetes. The mutated B7-1 molecule binds to the negative T cell regulator CTLA4, but not CD28. Co-delivery of the plasmid encoding the PPINS-GAD65 fusion structure and B7-1WA has a protective effect on insulitis and diabetes. In adoptive transfer experiments, DNA vaccination produced protective CD4^+^ Treg with a CD25^+^ or CD25 phenotype. In addition, the number of T cells with TR-related markers, such as CTLA4, Foxp3, and membrane-bound TGF-β, increased in the vaccinated mice. At the same time, CD4^+^ regulatory T cells (Tr) can inhibit the response of T cells to islet antigen (147). Yoshida et al. constructed DNA plasmid vectors that encode membrane binding to self-antigen proinsulin (MB-PPI) and PPI/GAD65w (GAD65 total protein) respectively. He injected mB-PPI and PPI/GAD65wDNA plasmid vectors together with MB7.1-IGG1/CD40L into female NOD mice. Five injections of DNA were given every three weeks. A significant delay and reduction in the incidence of autoimmune diabetes was observed. *In vitro* experiments showed that the spleen cells of protected mice did not produce increased IFN-*γ* when stimulated by insulin and GAD65 peptide (148). Monoclonal antibodies using CTLA4 usually have a masking effect, replacing a single amino acid in B7-1 (W88>A; B7-1wa) does not bind to CD28 or CTLA4. A plasmid encoding B7-1 or B7-1wa was constructed as a cell surface or Ig fusion protein. In the binding state, B7-1-IG enhanced CD3-mediated T cell activation, but B7-1wa-Ig had an inhibitory effect consistent with CTLA4 ligand. The B7-1wa (membrane-bound form) plasmid was combined with the PPINS plasmid, and the gene transfer was amplified by electroporation, and co-injected into non-obese autoimmune diabetic mice. Co-delivery of B7-1wa and PPINS cDNA eliminates insulin responsiveness and improves diabetes. Injection of either plasmid alone could not suppress the immune cell response to itself (149). In addition, Chang et al. designed a DNA vaccine combined with membrane-bound preproinsulin (mbPPI) and mB7.1/CD40L. The vaccine is able to promote the effective transmission of the autoantigen PPI, and its mutation B7.1 binds to CTLA4 but not CD28, well preventing cell recognition and killing by T cells (150). Patients with chronic T1D and the HLA-DRB1-0401 genotype received subcutaneous injections of proinsulin peptide epitopes (C19-A3) limited by the human leukocyte antigen DR4 (HLA-DR4). At various doses, IL-10-secreting peptide-specific T cells were seen, but proinsulin-specific pro-inflammatory T cells and systemic hypersensitivity were not observed (151).

#### 6.2.2 Polypeptide

In obese diabetic mice, Sema3E induces plexinD1-positive inflammatory macrophages into visceral white adipose tissue to regulate their biological defects. Yoshida et al. selected two antigenic peptides to produce neutralizing antibodies against amino acids 385-394(KVNGGKYGTT) or 359-368(HKEGPEYHWS) of Sema3E. The Ten terminus or lysine of each candidate peptide was conjugated by glutaraldehyde to the keyhole limpet hemocyanin (KLH) and given to obese wild-type male mice with Freund’s adjuvant. Vaccination with KLH-conjugated HKEGPEYHWS (Sema3E vaccine) significantly increased Sema3E antibody titer by screening. Sema3E vaccine inhibited plexind1-positive cell swelling, improved chronic inflammation in visceral white adipose tissue, and improved systemic glucose tolerance in obese mice (148). Consider that small interfering RNA (siRNA) was hindered by low transfection efficiency *in vivo*, Leconet et al. found that lipids or polyethyleneimine delivery agents can effectively treat siRNA molecules in pancreatic associated APCs. In terms of treatment, he found that short-term treatment with the lipid/ALOX15-specific siRNA complex promoted long-term protection against T1D in young wild-type (WT) NOD mice. In pancreatic associated CD11b^+^ cells, the PD-L1 pathway is upregulated and Treg are increased (152). β_2_ macroglobulin (β_2_m) was a universal signal component of MHC-I molecules when fused with cd3-Z chain. Therefore, the design of connecting the H-2KD binding insulin B chain peptide insulin B chain, amino acid 15-23 (IbsB_15-23_) to the N-terminal of β_2_m/CD3-ζ, made the mRNA encoding chimeric MHC-I receptor could target effector CD8 to diabetic CD8^+^ T cells, which reduced pancreatitis in NOD mice (153). Meanwhile, M2-polarized bone marrow derived macrophages (BMDMs) can also be used to secrete exosomes (Exos) containing miRNA to inhibit inflammation and maintain immune tolerance. After injection of M2 BMDM Exos in obese mice, miRNA consumption in exosomes blocked the ability of M2 BMDM Exos to enhance insulin sensitivity. Further analysis of miRNA showed that miR-690 was highly expressed in exosomes, and both *in vivo* and *in vitro* experiments proved that miR-690 targets NAD kinase (Nadk) and plays an important role in regulating macrophage inflammation and insulin signaling. Therefore, miR-690, as an insulin-sensitized miRNA, can be further explored (154). HSP60 peptide DiaPep277 is recognized to prevent adult humans and NOD mice from developing diabetes-related cell function decline. However, when the insulin dose and the level of glycosylated hemoglobin were treated in children with early-onset T1DM, there was no distinction between the patients in the control group and those in the blank control group. DiaPep277 thus does not maintain cell function (155, 156). Therefore, designing vaccinations to shield β-cells from damage now presents a new challenge.

#### 6.2.3 GAD65

Young NOD mice exhibited spontaneous Th1 response to glutamic acid decarboxylase (GAD65), which resulted in T cell autoimmunity. In order to prevent the autoimmunity before it happens naturally, Tian et al. introduced the notion of inducing active tolerance through the involvement of a Th2 immune response on GAD65 and testing if the T1D caused by series of Thl reactions can be prevented. As a result, young NOD mice were given a single intranasal injection of the GAD65 polypeptide, allowing for the detection of the high level of GAD65 IgGl antibody expression in the mice. GAD65 peptide administered intravenously reduced IFN-γ elevated IL-5 response, and promoted Th1 response with Th2 phenotypic transfer, decreased the prevalence of long-term T1D and insulitis (157). Agardh et al. evaluated the safety of alum formula for human recombinant GAD65 after suggesting an enhancement to its GAD65 polypeptide. Subcutaneous injections of various dosages of the modified vaccination did not significantly alter c-peptide levels or HbA_1c_ in persons with underlying autoimmune diabetes mellitus (LADA), whether fasting or hyperglycemic stimulation. It has been established that adding alum is a good way to enhance the GAD65 polypeptide vaccination (158). Moreover, GAD65 could also be packaged into vesicles to regulate hBA1c levels through nerve stimulation (159). Fengchun Li et al. modified the syngeneic splenocytes, and used retrovirus particles to transfer the gene encoding GAD65 into the spleen cells of NOD mice to make a lymphoid vaccine. A few weeks after the vaccine was injected into four-week-old NOD mice, high doses of the vaccine were found to reduce both insulitis and blood sugar levels (149). In addition, anti-CD3/GAD65 combination to treat T1D. The results showed that the amplification of GAD65-specific Treg was related to the mouse genotype. This provides further insights into the use of immunotherapy in the treatment of T1D. To improve the therapeutic effect, targeted individual immune monitoring is needed (160).

### 6.3 Cytokines

Liposomes, nanoparticles and other inorganic materials show certain advantages and are widely used in the treatment of tumor diseases (161). Treatment of tumors requires breaking immune tolerance, while for T1D, it is to restore immune homeostasis and protect β-cells. Therefore, inorganic materials can also be used as adjuvants to enhance the therapeutic effect of vaccines.

Yoon et al. has developed a vaccine-based approach using two synthetic controlled-release biomaterials, poly (lactate-co-polycerids; PLGA) microparticles (MPs) encapsulate denature-resistant insulin and PuraMatrix™ peptide hydrogel containing granulocyte macrophage colony stimulating factor (GM-CSF) and CpGODN1826 (CpG). Interestingly, three subcutaneous injections of this hydrogel (GM-CSF/CpG)/insulin-MP vaccine protected most NOD mice against T1D infection. At the same time, the stimulating effect of CpG increased IL-10 production. Multiple injections of insulin-containing agents under the skin can form granulomas, creating a kind of microenvironment that recruits immune cells. Accordingly, this injectable hydrogel/MP based vaccine system make it possible to prevent T1D with (162). Based on inhibition of autoreactive T cells by Foxp3^+^ Treg, impaired tolerance promotes the destruction of insulin-producing β-cells in autoimmune T1D. So Hyoty and Knip treated human hematopoietic stem cell transplanted NG-HLA-DQ8 transgenic mice with upon subimmunogenic vaccine. It was observed that the hypoimmunogenicity boosted the levels of insulin-specific Foxp3^+^ Treg in the human immune system. Moreover, the expression of Foxp3, CTLA4, IL-2RA and TIGIT increased, which effectively inhibited effector T cells. Therefore, inducing the expression of highly efficient human insulin-specific Foxp3^+^ Treg *in vivo* can be the direction of new human insulin mimics (163). Interleukin-1β (IL-1β) is a key cytokine involved in inflammatory diseases, which can be treated from the perspective of decreased IL-1β activity. Cavellti-Weder et al. synthesized a novel vaccine against IL-1β. The vaccine hIL1bQb consists of full-length, recombinant IL-1β coupled virus-like particles. IL-1β-specific antibodies were induced immediately after vaccination in preclinical apes, while neutralizing antibodies were delayed. In a clinical study of 48 patients with type 2 diabetes, neutralizing IL-1β specific antibody responses were detected after multiple injections of anti-IL-1β vaccine. The patient’s blood glucose and body weight were improved without obvious adverse reactions (164). In addition, Spohn et al. chemically cross-conjugated IL-1β with virus-like particles of phage Qβ to form a detoxifying version of the IL-1β vaccine. The vaccine was well tolerated in mice and neutralized the biological activity of IL-1β. Nor did infecting mice with Listeria monocytogenes or Mycobacterium tuberculosis induce an immune response. And the IL-1β vaccine also improved glucose tolerance in diet-induced type 2 diabetes (165). In addition, DCs expressing interleukin 4 (IL-4) were induced by lentiviral transduction. To avoid the dual risk of carcinogenicity or immune-heredity of cell modification with viral vectors, DCs were used electrocuted to “translation-enhanced” of IL-4mRNA (eDC/IL-4) in NOD mice, and demonstrated that electroporation did not affect the therapeutic effect. After a single injection of eDC/IL-4 shortly after the onset of hyperglycemia in NOD mice, some mice could maintain blood glucose stability for several months. The eDC/IL-4 treatment enhanced the function of Treg and modulated T-assisted responses to reduce pathogenicity (166). Microcarriers are known to direct DCs to the site of administration and, once phagocytic, the contents can form a DC phenotype. Therefore, promaxtm microspheres were proposed to form antisense oligonucleotides and enable autologous dendritic cells to inhibit diabetes. When subcutaneously injected into NOD mice, the vaccine enhanced the expression of CD25^+^Foxp3^+^ Treg. *In vitro* experiments also demonstrated that the vaccine could inhibit NOD-derived pancreatic cell antigen activity (167). Au et al. synthesized β-cells with high expression of PD-L1, CD86 and GAL-9 and subcutaneously injected β-cells with high express co-inhibitory immune checkpoint ligands. It can induce islet antigen specific immune tolerance to autoreactive T cells and reverse early onset hyperglycemia (168).

The vaccination has demonstrated some benefits in the management of diabetes, starting with bacteria, insulin, β-cells, and GAD65, though diabetes patients could not be fully treated for a variety of reasons. Therefore, in order to maintain autoimmune tolerance, it is important to decide whether to combine a vaccine with immunological checkpoint or anti-inflammatory agents, as well as to manufacture insulin to maintain normal blood glucose levels.

## 7 Conclusion and perspective

In summary, as we have review above, there are three aspects for the treatment of new-onset diabetes by immune modulation. Firstly, for immunosuppressive factors, the mostly employed ones are TGF-β, IL-2, etc. Currently, organic synthetic materials, such as PLGA and PEGylated, have been used to encapsulate immunosuppressive factors to achieve sustained drug release to control blood glucose. Alternatively, nanoparticles or device reduce the toxic side effects and enhance efficacy of the immunosuppressive drugs. In addition to the current approaches, it can use cell membrane vesicles to encapsulate the epidemic suppressor. Secondly, the most common and promising treatment for T1D is the vaccine. To prevent T1D, the vaccines based on virus or bacteria that elicit T1D can be injected into the body in advance. There are many therapeutic vaccines available even the patients have with T1D. It is common to use proinsulin as a vaccine, or the insulin peptide also could be wrapped in liposomes or other biological materials as nanoparticles vaccines, that facilitate the capture by DC. However, for patients whose islets are not completely damaged, how to restore immune tolerance should be considered. GAD56 is another common vaccine candidates, which can robustly induce Treg cells to play the immunosuppressive role. Mixing a variety of immunosuppressive factors together with vaccines is a promising therapeutic strategy for T1D therapy. For T1D vaccine, enhance of the immunogenicity of the antigen is a promising way. As for adjuvants, adjuvant vaccines are extremely important. Bacterial membrane derived vesicles had been intensively exploring as cancer vaccine adjuvant that can also are be considered for enhancing immunogenicity of the T1D antigens. For example, proinsulin peptide vaccines can be coated with bacterial membranes to promote the robust immune response. Thirdly, engineered cell-based therapy such as Tregs, MDSC, M2 macrophages that could be employed to restore the immune tolerance. Cells could be isolated from patients and proliferated *in vitro* based on specific amplification or engineering approaches to maintain immune tolerance, then transfuse to individual to treat T1D. We envision that the future applications of immunosuppressive cells could be in combination with immunosuppressive agents’ target delivery or immune checkpoint blockade of islet-autoreactive T cell to prevent its pathological killing of β-cells. Alternatively, inflammatory suppressive cytokines could be overexpressed in immunosuppressive cells, whose exosomes could be extracted to treat T1D together with insulin.

Together, combining biomaterials to form drug delivery nanoparticles or device holds great potential for treating T1D, and further research and exploration in the delicate design of biomaterials and unknown mechanistic signaling pathways would make a huge contribution.

## 8 Future and challenges

The particularity of biomaterials can achieve some purposes of T1D treatment. Treating T1D from an immunological perspective has many merits. Firstly, immune checkpoint modulation is one of the most promising ways in protecting β-cells from T cell attack. At present, PD-1/PD-L1, CTLA4, GAL3, IDO and other checkpoints have been paid more and more attention in cancer immunotherapy. Except these above, the ICOS/ICOSL signaling deserves attention. It has shown potent antitumor activity in combination with CTLA4 blockers in tumor immunotherapy (69). These immune suppressive legends such as PD-L1, Gal-9 and CTLA4 could be engineered to treat T1D through reactively inhibit the T cell activity. In addition, these negative immune checkpoint proteins also be wrapped exosome, platelets, hydrogels and other nanoparticles to inhibit T cells to protecting β-cells. However, how to target delivery these native immune checkpoint proteins to pancreas and reduce their off-target immune suppression is still a challenge.

Secondly, for vaccines, the preventive and therapeutic functions cannot be ignored, though the induced immune response rate is relatively low by current antigen candidates. The components of a vaccine are mainly divided into two parts, antigen and adjuvant. To improve the effect, it can not only improve the immunogenicity of autoantigen, but also adjust the adjuvant to cause autoimmunity. Since EMC-D can rapidly destroy β-cells by replicating in cells, it is considerable to modify the structure of EMC-D to remove pathogenic toxicity and act as an antigen to cause immune response at the same time, so as to prevent T1D. In addition, whether bacterial membrane vesicle can be engineered to contain multiple surface proteins to inhibit inflammation is worth considering. For example, how about inhibiting the expression of IL-1β, IL-6, IL-10 simultaneously?

Thirdly, referred to immunosuppressive cells, *in vitro* expansion has achieved impressive success and numerous clinical trials were ongoing, especially in Treg. During this process, more efficient cell types were detected, such as antigen-specific Treg, which encourages researchers to explore new ways to achieve it. So far, major challenges are summarized as follows: 1) Optimized separation and amplification process of specific cells; 2) limited expandability based on the patient’s condition; 3) Further confirmation of efficacy and mechanism of engineered immunosuppressive cells.

Hence, challenges to biomaterials applied in T1D still persist today. However, we envisage that employing biomaterials to facilitate the treatment of T1D based on immunotherapy is promising.

## Author contributions

ZJ, YL, YM contributed equally to this work. XiZ, ZJ, YL, YM wrote the manuscript. XuZ, ZJ, XiZ helped to revised the manuscript. The manuscript was written through contributions of all authors. All authors have given approval to the final version of the manuscript.

## Funding

This work was supported by the National Natural Science Foundation of China (31971268, 32201084), grants from Science, Technology & Innovation Commission of Shenzhen Municipality (JCYJ20200109142610136, JCYJ20180507181654186), Guangdong Basic and Applied Basic Research Foundation (2019A1515010855), Guangdong Basic and Applied Basic Research Foundation (2020A1515110166), the Natural Science Foundation of Guangdong Province (No. 2020A1515010802, No. 2022A1515012289), Shenzhen Science and Technology Program (Grant No. RCYX20200714114643121), University of Chinese Academy of Sciences-Shenzhen Hospital Research Funding (HRF-2020004), the Health system scientific research project of Shenzhen Guangming District Science and innovation Bureau (2020R01073, 2020R01061), Special fund for economic development of ShenZhen Guangming District (2021R01128), Doctoral personnel scientific research start-up Fund project of Guangdong Medical University (GDMUB2022037), Fundamental Research Funds for the Central Universities (19lgzd45), and the Health system scientific research project of Shenzhen Guangming District Science and innovation Bureau (2020R01073, 2020R01061).

## Acknowledgments

Xudong Zhang, Pingping Li, Wen-I Yeh, Alessia Zoso, Roham Parsa, Kin Man Au, Katja Stifter, Wilhem Leconet at al. for improvement.

## Conflict of interest

The authors declare that the research was conducted in the absence of any commercial or financial relationships that could be construed as a potential conflict of interest.

## Publisher’s note

All claims expressed in this article are solely those of the authors and do not necessarily represent those of their affiliated organizations, or those of the publisher, the editors and the reviewers. Any product that may be evaluated in this article, or claim that may be made by its manufacturer, is not guaranteed or endorsed by the publisher.
